# Comparison of SCORE, SCORE2 and Framingham Risk Score-Based Methods for Vascular Age Calculation

**DOI:** 10.3390/jcm14217570

**Published:** 2025-10-25

**Authors:** Helga Gyöngyösi, Beáta Kőrösi, Dóra Batta, Zsófia Nemcsik-Bencze, Andrea László, Péter Torzsa, Dániel Eörsi, Johanna Takács, János Nemcsik

**Affiliations:** 1 Department of Family Medicine, Semmelweis University, 1085 Budapest, Hungary; gyongyosi.helga@semmelweis.hu (H.G.); beataz.korosi@gmail.com (B.K.); batta.dori@gmail.com (D.B.); torzsa.peter@semmelweis.hu (P.T.); danieorsi@gmail.com (D.E.); 2 Neurocognitive Research Centre, Nyiro Gyula National Institute of Psychiatry and Addictology, 1135 Budapest, Hungary; zsofia.nemcsik@gmail.com; 3 GP Practice Jula/Schindler, 90480 Nuremberg, Germany; laszloandrea@gmail.com; 4 Faculty of Health Sciences, Semmelweis University, 1085 Budapest, Hungary; takacs.johanna@semmelweis.hu

**Keywords:** vascular age, risk scores, SCORE, SCORE2, Framingham Risk Score

## Abstract

**Background/objectives:** Calculation of vascular age can help patients to understand the importance of adherence to a healthy lifestyle and medications. There are different methods of calculating vascular age, but different methods can provide different vascular age results. Our aim was to evaluate vascular age based on the Systematic Coronary Risk Evaluation (SCORE), Systematic Coronary Risk Evaluation 2 (SCORE 2) and Framingham Risk Score (FRS) methods. **Methods:** Subjects between the ages of 40–65 were involved. Vascular ages were defined based on SCORE, SCORE2 and FRS methods according to data from the literature. **Results:** In total, 141 patients were involved in the study; among them 94 had hypertension (HT) and 23 had white-coat hypertension. In the total population, SCORE2 and FRS vascular ages were higher compared to chronological age. SCORE2 and FRS vascular ages were higher than SCORE vascular age, and FRS vascular age was higher compared to SCORE2 as well. These tendencies were the same in the case of hypertensive patients and in patients with white-coat hypertension. In healthy patients, there were no differences between chronological age and vascular age. **Conclusions:** The differences found between the calculated vascular ages and the proportion of subjects with elevated vascular age warrant further comparison of different vascular age calculation methods.

## 1. Introduction

Cardiovascular (CV) diseases are the primary global cause of morbidity and mortality, leading to 17.9 million deaths in 2018, as reported by the Global Burden of Disease Study [1]. Lowering blood pressure and cholesterol levels significantly reduces the risk of developing CV diseases and mortality in various patient groups [2,3]. On the contrary, non-adherence to medications can lead to an increased prevalence of CV events [4].

Communicating CV risk can motivate patients toward healthier lifestyles and medication adherence. However, presenting absolute CV risk as a percentage may not always be convincing due to potentially low numerical values and the risk of misinterpretation. The vascular age concept offers a more impactful approach, illustrating whether a patient’s vascular system is older than their chronological age, which might significantly improve long-term adherence to preventative strategies [5]. In physiological terms, vascular age reflects the biological condition of blood vessels, which often deteriorate faster than chronological age due to factors like oxidative stress, chronic inflammation, and endothelial dysfunction. These changes lead to arterial stiffening and calcification, increasing the risk of CV diseases [6].

The Systematic COronary Risk Evaluation (SCORE) was a widely used tool, particularly in Europe, for over 10 years to estimate the 10-year risk of fatal CV events. It remained popular until SCORE2 was introduced in 2021 [7,8]. Vascular age calculation based on SCORE was developed in 2010 [9].

Compared to SCORE, SCORE2 introduced several key updates in 2021, when it was introduced in the CV prevention guidelines: it uses non-HDL cholesterol instead of total cholesterol for risk estimation, the age range was extended up to 69 years, and an additional dedicated chart was created, called SCORE2-OP, for individuals aged 70–89 years. Additionally, SCORE2 considers both 10-year fatal and non-fatal CV events, leading to adjusted percentage classifications for risk categories and different ranges across various age groups compared to SCORE. Vascular age calculation is also available in this guideline, named “risk age”, and is automatically calculated online as part of the HeartScore calculator [7,10].

The Framingham Risk Score (FRS) is a widely recognized method, particularly in the United States, for stratifying CV risk. This score, published in 2008, was developed using data from 8.491 participants in the Framingham Study [5]. The same study that introduced the FRS also contained the concept of vascular age [5].

The importance of risk calculation and communication is inevitable. Risk calculation scores are essential tools for physicians, as they provide an evidence-based, standardized framework for individualized patient risk stratification and guiding the intensity of preventive treatment according to clinical guidelines. These methods provide a quantifiable number that helps patients to understand their individual risk. This might be a powerful motivator for lifestyle changes and medication adherence.

Previously we demonstrated that vascular ages calculated by SCORE or FRS provided markedly different values and identified different patients with elevated vascular age [11]. However, no comparison is available in the literature that considers the vascular age based on SCORE2 in such a setting. The aim of the study was to evaluate and compare vascular ages based on SCORE, SCORE2 and FRS and to compare patient characteristics with elevated vascular ages identified by different methods.

## 2. Methods

Between August 2012 and January 2019, a cross-sectional CV screening study was conducted involving Caucasian individuals aged over 18 years from three general practitioner practices in Budapest, Hungary. In the present study, healthy individuals, patients with white-coat hypertension and patients with chronic non-resistant or chronic resistant hypertension without history of CV disease between the ages of 40–65 were involved. Subjects with dementia were excluded, the condition potentially impairing the completion of an auto-administered questionnaire. Subjects with atrial fibrillation were also excluded, as all subjects had tonometric arterial stiffness measurements as well—data which are not shown in the present study. Patients with diabetes were also excluded, as SCORE2 can not be applied to them. Some findings from this cohort have been published [11,12]. During the screening visit, participants completed a self-administered questionnaire covering personal and family medical history. They were then scheduled for a clinical appointment (7:00–8:00 a.m.) involving office brachial blood pressure measurement and blood sampling. Cardiovascular vascular age was calculated after laboratory results became available. All subjects provided written informed consent prior to participation. The study received approval from the Scientific and Research Ethics Committee of the Medical Research Council, Hungarian Ministry of Health (ETT TUKEB 842/PI/2011), and was conducted in accordance with the Declaration of Helsinki.

### 2.1. Evaluation of Office Blood Pressure and Definition of Hypertension Phenotypes

On the morning of clinical evaluation, following five minutes of rest, blood pressure was recorded twice on each arm using a validated oscillometric device (Omron M3, Kyoto, Japan). The highest average value from either arm was used for analysis. Hypertension was classified in accordance with the recent European guidelines [13]. White-coat hypertension was characterized by elevated office blood pressure (>140/90 mmHg) alongside normal readings on 24 h ambulatory blood pressure monitoring (ABPM): average ≤ 130/80 mmHg, daytime ≤ 135/85 mmHg, and nighttime ≤ 120/70 mmHg. Resistant hypertension was defined by persistently high office blood pressure (>140/90 mmHg) despite the use of three antihypertensive drugs from distinct classes—including a diuretic—or controlled blood pressure requiring more than three medications [14]. Chronic non-resistant and chronic resistant hypertensive patients were analyzed in a merged group as “hypertensive patients”.

### 2.2. Calculation of Vascular Age Based on Framingham Risk Score (FRS)

Vascular age was estimated using the Framingham Risk Score (FRS) method as described in the original publication by D’Agostino et al. [5]. This method incorporates age, total cholesterol, HDL cholesterol, brachial systolic blood pressure, current antihypertensive treatment, smoking status and presence of diabetes, with gender specific outcomes. The vascular age approximation involves first calculating the individual’s FRS-based CV risk, then identifying the age of a person with the same predicted risk but with all other risk factors within normal limits [5]. Although the FRS tool defines the highest possible vascular age as ‘80+’, an age of 80 years was used for all individuals exceeding this threshold in mathematical calculations.

### 2.3. Calculation of Vascular Age Based on Systematic COronary Risk Evaluation (SCORE) Risk Score

The SCORE risk calculation incorporates age, sex, brachial systolic blood pressure, total cholesterol, and smoking status, with different risk charts applied for low- and high-risk European countries [8]. Vascular age estimation using the SCORE method follows the same principle as the FRS-based approach: it determines the age at which an individual would have the same estimated CV risk if all risk factors were within normal limits [9]. A high degree of consistency between vascular age values derived from both SCORE charts indicates this method’s wide applicability across European risk settings [9].

### 2.4. Calculation of Vascular Age Based on Systematic Coronary Risk Evaluation 2 (SCORE2)

The SCORE2 risk calculator is an upgraded version of the SCORE, published in 2021 [7]. The calculator is based on age, sex, brachial systolic blood pressure, non-HDL cholesterol and smoking status and predicts the 10-year risk of both fatal and non-fatal CV events. For vascular age calculation, an online HeartScore calculator is available [10]. This calculator was used manually in our study.

Elevated vascular age was defined based on previous publications [11,15] where the age difference was ≥2 years between the chronological age and the vascular ages.

### 2.5. Statistical Analysis

Descriptive data are expressed as mean ± standard deviation or median with interquartile ranges or percentages. The normality of continuous parameters was tested with the Kolmogorov–Smirnov test. For the comparison of the chronological age and vascular ages calculated with FRS, SCORE or SCORE2, ANOVA and Kruskal-–Wallis H tests were used in the whole cohort and in the studied subgroups as well (patients with hypertension, patients with white-coat hypertension and healthy patients). Logistic regression analyses were performed to identify the determinants of elevated vascular age. A *p* < 0.05 was considered to be significant. SPSS 27.0 for Windows (IBM Corp., Armonk, NY, USA) was used for all calculations.

## 3. Results

One hundred and forty-one (141) patients were involved in the study. Among them, 94 had hypertension (chronic resistant hypertension and chronic non-resistant hypertension) and 23 had white-coat hypertension. Table 1. shows the baseline characteristics and laboratory and hemodynamic parameters in the total group and in subgroups, where the vascular age was higher than the chronological age with the three used methods (SCORE+, SCORE2+, FRS+). The chronological age and the vascular ages calculated by the three methods were higher in the SCORE+ group compared to the SCORE2+ and FRS+ groups. A lower proportion of smokers was in the FRS+ group (28.6%) compared to the SCORE+ (66.7%) and SCORE2+ groups (57.1%).

**Figure 1:**
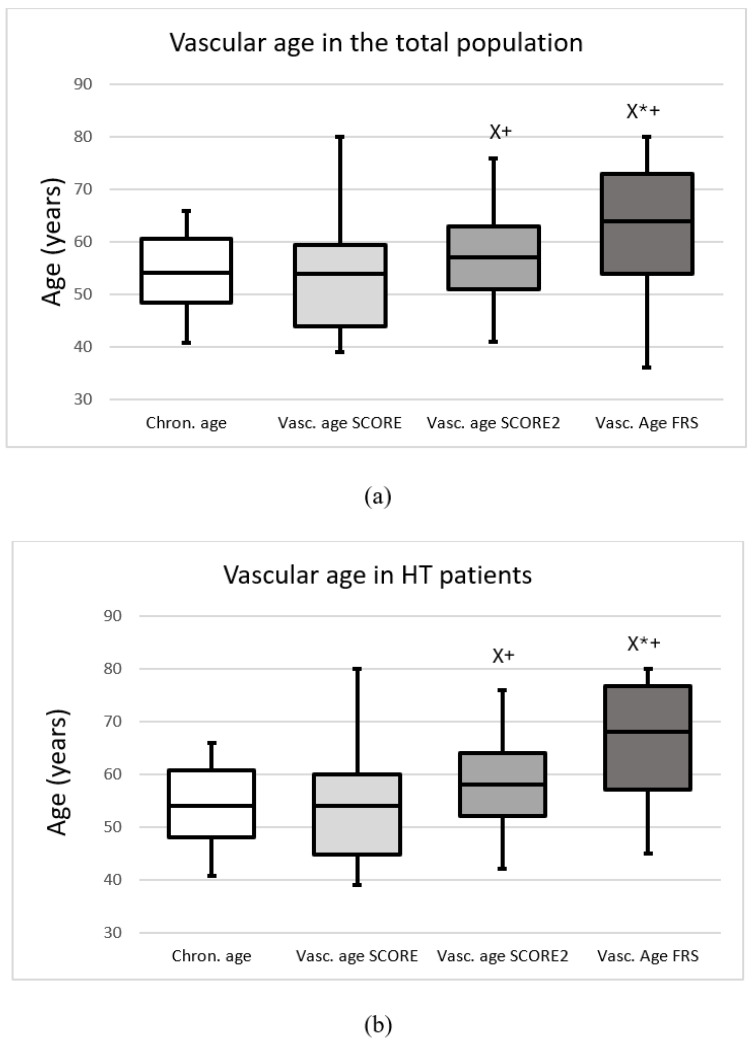

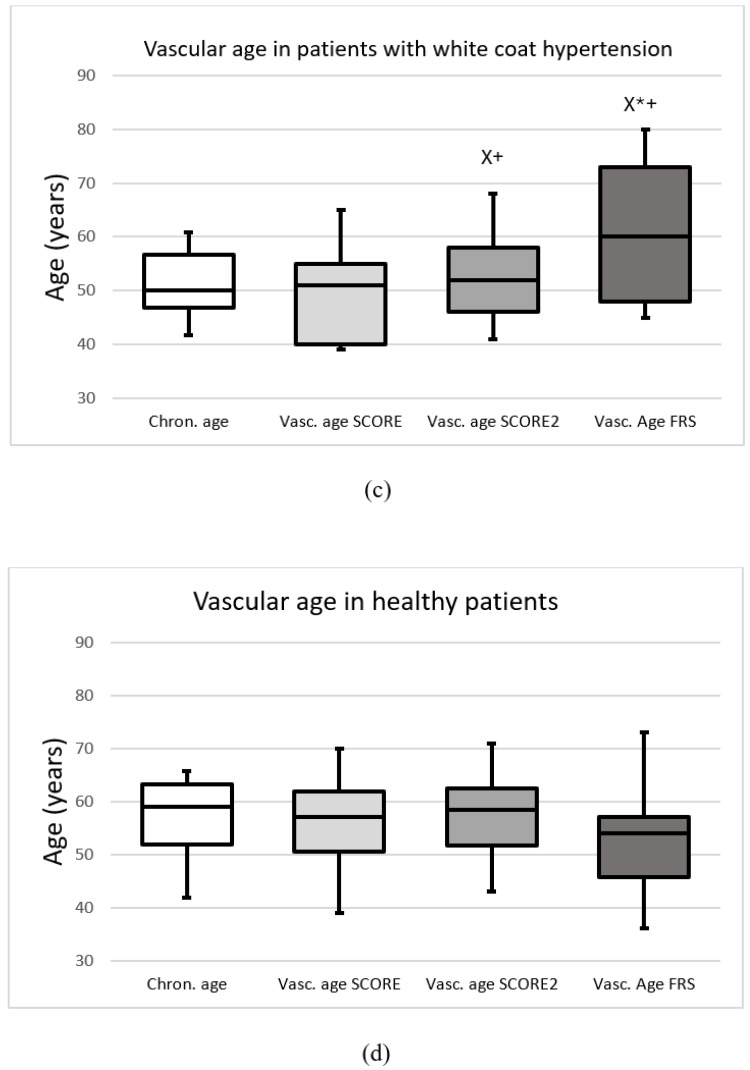
The chronological and vascular ages calculated with the Systematic Coronary Risk Evaluation (Vasc. age SCORE), Systematic Coronary Risk Evaluation 2 (Vasc. age SCORE2) and Framingham Risk Score (Vasc. age FRS) (**a**) in the total population, (**b**) in patients with hypertension, (**c**) in patients with white-coat hypertension, (**d**) in healthy patients. Data are presented as the median (interquartile ranges in columns and minimal, maximal values in error bars). X significant difference compared to SCORE; * significant difference compared to SCORE2; + significant difference compared to chronological age (*p* < 0.05).

The median chronological age of the total population was 54.2 (48.5–60.5) years, while SCORE, SCORE2 and FRS vascular ages were 54.0 (44.0–59.5), 57.0 (51.0–63.0) and 64.0 (54.0–73.0) years, respectively. In hypertensive patients, chronological, SCORE, SCORE2 and FRS vascular ages were 53.9 (47.9–60.75), 54.0 (44.75–60.0), 58.0 (52.0–64.0) and 68.0 (57.0–76.75) years, respectively (*p* < 0.05). In patients with white-coat hypertension, chronological, SCORE, SCORE2 and FRS vascular ages were 50.1 (46.8–56.7), 51.0 (40.0–55.0), 52.0 (46.0–58.0) and 60.0 (48.0–73.0) years, respectively (*p* < 0.05). Figure 1 demonstrates the chronological and vascular ages calculated with the SCORE, SCORE2 and FRS methods in the total group (a), in HT patients (b), in WhHT patients (c), and in healthy patients (d).

We investigated, using logistic regression analysis, whether certain variables not accounted for by the risk score systems (positive family history, uric acid, BMI) are independent determinants of elevated vascular age as assessed by various methods. We found that elevated uric acid level is a predictor of increased vascular age in the case of FRS (B = 0.006; SE = 0.002; *p* = 0,020; OR = 1.006 [95%CI:1.001–1.010]).

**Figure 2:**
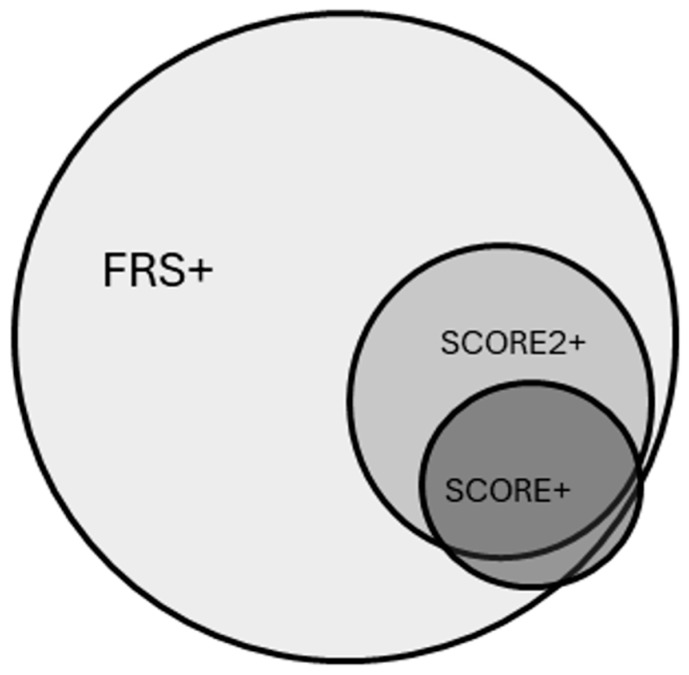
Overlap between subjects with elevated vascular age having at least 2 years of difference compared to their chronological age based on the Framingham Risk Score-based method (FRS+), the SCORE-based method (SCORE+), or SCORE2-based method (SCORE2+).

In the total population, SCORE2 and FRS vascular ages were higher compared to chronological age. SCORE2 and FRS vascular ages were higher than SCORE vascular age, and FRS vascular age was higher compared to SCORE2 as well. These tendencies were the same in the cases of hypertensive patients and in patients with white-coat hypertension. In healthy patients, there were no differences between chronological age and vascular age. Based on SCORE, SCORE2 and FRS 15.7%, 35.1% and 68.1% of the subjects had increased vascular age compared with chronological age, respectively (*p* < 0.05). 14.9% (n = 23) of the patients had elevated vascular age with SCORE and SCORE2; furthermore, 31.8% (n = 49) of the patients had elevated vascular age with SCORE2 and FRS and 16.8% (n = 26) of the patients had elevated vascular age with SCORE and FRS. In addition, 14.9% (n = 23) of the patients had elevated vascular age with all the three methods. Figure 2 shows the overlap between subjects identified with elevated vascular age.

## 4. Discussion

This is the first study that has compared vascular ages calculated with three different risk score-based methods, including SCORE2. Differences were found between the vascular ages calculated with SCORE, SCORE2 and FRS in the total population and also in subjects with impaired vascular ages evaluated with the three methods. According to these findings, vascular ages differ across different patient groups. It is important that different methods identify the same patients as having elevated vascular age. However, based on our study, while there was an overlap among the three methods and SCORE2 attenuated the difference between SCORE and FRS, marked differences were still identified.

In our previous study, SCORE, FRS and carotid-femoral pulse wave velocity (cfPWV)- based vascular age calculation methods were compared. In line with our present results, differences were observed between the methods. The vascular age calculated with the FRS method was higher in patients with hypertension and diabetes; however, analyzing healthy and white-coat hypertensive patients, there were no differences in the calculated vascular ages. There was also a marked difference between the proportion of the patients identified with elevated vascular age with the three methods [11]. In a population-based study, which involved 99,320 patients who participated in a national screening programme in Hungary, similar results were observed in favour of FRS in patients with hypertension and diabetes [15]. In our present study, the SCORE2-based vascular age calculation method attenuated the previously found differences between SCORE and FRS, but FRS still identifies a higher proportion of patients with elevated vascular age.

Studies have already been conducted using SCORE2 as a risk stratification method. The study by Stömberg et al. found a clear association between increased cardiovascular risk, as assessed by the SCORE2 algorithm, and the presence of subclinical vascular damage, specifically increased arterial stiffness and significant coronary calcification, in a general population without diabetes or established cardiovascular disease. The prevalence of both types of vascular damage was highest in the very high SCORE2 risk group and lowest in the low-to-moderate risk group. Although there was a correlation, the overlap between arterial stiffness and coronary calcification was relatively modest, suggesting that PWV and CACS describe different types of vascular damage [16]. However, our results are the first in the literature which consider the SCORE2-based “risk age” in comparison with other vascular age methods.

Besides PWV as a measurement-based method to identify early vascular ageing, other device-based methods are also available. Our research team observed similar trends in vascular age differences in a study involving 241 participants, where coronary artery calcium score-based vascular age was assessed following coronary computed tomography angiography together with FRS- and SCORE-based vascular ages [17]. In individuals with diabetes and treated hypertension, our findings are also in line with those of Kozakova et al., who examined 528 patients [18]. In their study, early vascular ageing was defined using the distensibility coefficient of the common carotid artery, and results revealed that vascular age estimated by FRS was consistently higher than that which was calculated using the SCORE method.

The use of various vascular age assessment methods also indicates that vascular ageing occurs through multiple pathophysiological pathways. There are both functional and structural implications of changes in the wall of the blood vessel. Functional ageing is mainly manifested in endothelial dysfunction, with a decrease in nitric-oxide production. Increased collagen production, collagen crosslinking, reduction, fracture and calcification of elastin cause structural changes in larger elastic arteries. There is also an increase in vascular smooth muscle growth, which contributes to thickening of arterial walls and an increase in arterial stiffness [19]. According to the recent study by Madaudo et al., dysfunctional HDL cholesterol also contributes to the development of cardiovascular diseases through multiple mechanisms. These include reduced activity of paraoxonase 1 and glutathione peroxidase, which lead to increased oxidative stress and promote the formation of oxidized LDL cholesterol, both of which are critical in the pathogenesis of atherosclerosis and accelerated vascular ageing [20]. These complex mechanisms highlight that using different risk scores to approximate vascular age captures only a portion of the overall vascular ageing process.

We suppose that using SCORE2 instead of SCORE to calculate vascular age reduces the magnitude of the difference with FRS because of the methodological changes. A main difference between SCORE and SCORE2 is that similarly to FRS, SCORE2 considers non-lethal CV events as well, while SCORE only estimates the probability of fatal CV events. Due to this change, SCORE2 estimation may approach the FRS calculation. On the other hand, there are still differences between the number of patients and the overlap between patients identified with elevated vascular age between the three different methods, so the use of SCORE2 only mitigates the previously observed difference between SCORE- and FRS-based vascular age calculation methods.

These findings suggest that among patients with treated hypertension and white-coat hypertension, calculating vascular age using FRS might be more effective in communicating CV risk compared to the SCORE and SCORE2 methods. However, it is important to note that this is only a hypothesis, and follow-up studies are needed to validate this suggestion. Identifying individuals whose vascular age exceeds their chronological age is vital for initiating more intensive preventive strategies that go beyond pharmacological blood pressure management. Such interventions may include lifestyle modifications like increased physical activity [21], dietary changes [22], and reduced salt intake [23,24] as well as strict monitoring of medication adherence. It is also important to communicate the CV risk, even in patients with established diagnosis of hypertension. Van Wijk et al. conducted a retrospective cohort study using Dutch pharmacy and hospital records to assess long-term persistence with antihypertensive therapy. Among 2 325 patients who initiated treatment in 1992, only 39% remained continuously adherent over a 10-year period and 22% temporarily discontinued and restarted treatment [25]. According to an online survey that provides participants with the same hypothetical CV risk in different ways, using vascular age led to improvements in several aspects of risk understanding and intentions to take actions independently of their cognitive skills. The inclusion of vascular age also positively affected motivation to visit the GP for further screenings [26]. On the other hand, failing to identify these at-risk individuals may result in missed opportunities for lifestyle-based prevention, potentially compromising long-term health outcomes. In our cross-sectional study, we highlighted the differences between vascular age calculation methods; however, longer follow-up studies are needed to confirm our hypothesis regarding the clinical importance and impact on outcomes of the differences between these methods in both healthy and hypertensive patients.

A significant challenge requiring consensus is the precise definition of early vascular ageing based on calculations of the difference between chronological age and vascular age. There is currently no standardized threshold for this difference. For example, some studies, such as the one by Antza et al. [27], compared a newly constructed early vascular ageing identification score against a cfPWV value that was simply above the age-adjusted mean. In contrast, Nedogoda et al. [28] established a stricter definition, classifying early vascular ageing as cfPWV values that exceed the expected age-matched values by at least two standard deviations. Meanwhile, Nilsson et al. [29] used a percentile-based approach, defining early vascular ageing subjects as those falling within the highest 10% of the standardized cfPWV distribution for their respective age intervals.

This study has some limitations. First, there is no clear guideline for a vascular age cut-off that would indicate the need for lifestyle changes. We had to arbitrarily choose a 2-year difference as our threshold, which is not a widely accepted value; however, we used this threshold previously in a publication [15]. The small sample size, the heterogeneity of our study group and the low general CV risk profile of the participants limit the generalizability of our findings. According to the ESC Guideline on Cardivascular disease prevention in clinical practice [7]. Hungary is considered as a high-risk country, and this fact also limits the generalizability of our results. Additionally, the cross-sectional design restricted our ability to perform more in-depth analysis.

## 5. Conclusions

We found that different score-based methods for calculating vascular age yield markedly different results. This variation directly affects which subjects are identified as having an elevated vascular age. Our results suggest that different vascular age estimation methods diverge across different patient groups, and our research raises the need for long-term follow-up studies to identify the appropriate risk-estimating scoring system for the patient, taking their underlying conditions into account.

## Figures and Tables

**Table 1:** Characteristics of the total population and subjects with elevated vascular age by different methods.

	Total Population	SCORE+	SCORE2+	FRS+
n	141	27 (19.1%)	49 (24.8%)	105 (74.5%)
Chronological age (years)	54.2 ±7.1	57.4 ± 6.1 *#	52.5 ± 7.3	53.6 ± 7.2
SCORE vasc.age (years)	53.1 ± 9.7	63.9 ± 7.7 *#	54.4 ± 11.6	53.1 ± 10.3
SCORE2 vasc.age (years)	56.7 ± 7.7	65.1 ± 6.1 *#	60.2 ± 8	57.1 ± 8.1
FRS vasc.age (years)	63.2 ± 11.9	75.9 ± 6.7 *#	70.8 ± 10	67.4 ± 10.4
SBP (mmHg)	125.5 ± 14.5	136.4 ± 19.2	134 ± 16.6	129.7 ± 14.1
HR (1/min)	67 ± 9	68 ± 15	69 ± 10	68 ± 10
BMI (kg/m^2^)	27.0 ± 4.5	27.7 ± 4.8	27.3 ± 4.5	27.3 ± 4.3
SeChol (mmol/L)	5.9 ± 1	6.3 ± 1.0	6.2 ± 0.9	6.0 ± 1.0
LDL-c (mmol/L)	3.8 ± 0.8	4.4 ± 0.9	4.1 ± 0.8	3.9 ± 0.8
HDL-c (mmol/L)	1.4 ± 0.4	1.5 ± 0.4	1.3 ± 0.3	1.4 ± 0.4
Triglyceride (mmol/L)	1.5 ± 0.7	1.5 ± 0.8	1.7 ± 0.8	1.6 ±0.8
GFR (mL/min/1.73 m^2^)	89.5 ± 14. 5	89.6 ±14.9	90.0 ± 17.8	90.0 ± 15.1
Uric acid (mmol/L)	314.1 ± 80.7	327. 9 ± 106.4	327.3 ± 89.9	322.8 ± 83.2
HT (n)	94 (65.7%)	22 (81.5%)	42 (85.7%)	82 (78.1%)
WhHT (n)	23 (16.1%)	3 (11.1%)	4 (8.2%)	18 (17.1%)
Smoking (n)	31 (21.7%)	18 (66.7%)	28 (57.1%)	30 (28.6%) *X
Family history of stroke (n)	44 (30.8%)	7 (26.9%)	14 (28.6)	32 (30.5%)
Family history of coronary heart disease (n)	43 (30.1%)	5 (18.5%)	11 (22.4%)	32 (32.4%)
Family history of sudden cardiac death (n)	22 (15.4%)	4 (14.8%)	4 (8.4%)	16 (15.2%)

SCORE+, subjects with elevated vascular age based on SCORE; FRS+, subjects with elevated vascular age based on the Framingham Risk Score method; SCORE2+, subjects with elevated vascular age based on SCORE2; FRS vasc. age: vascular age based on the Framingham Risk Score method; SCORE vasc. age: vascular age based on SCORE; SCORE2 vasc. age: vascular age based on SCORE2; BMI: body mass index; GFR-EPI: glomerular filtration rate assessed by the chronic kidney disease epidemiology collaboration glomerular filtration rate equation; Chol: total cholesterol; LDL-c: low density lipoprotein cholesterol; HDL-c: high density lipoprotein cholesterol. Data are presented as mean ± standard deviation. Categorical parameters are presented as n (%). Significant differences are formatted as bold and italic characters: X significant difference compared to SCORE; * significant difference compared to SCORE2; # significant difference compared to FRS.

## Data Availability

The original contributions presented in this study are included in the article. Further inquiries can be directed to the corresponding author.
